# Repurposing auranofin to treat *TP53*-mutated or *PTEN*-deleted refractory B-cell lymphoma

**DOI:** 10.1038/s41408-019-0259-8

**Published:** 2019-11-28

**Authors:** Jeffrey Wang, Jacqueline Wang, Elyse Lopez, Hui Guo, Hui Zhang, Yang Liu, Zhihong Chen, Shengjian Huang, Shouhao Zhou, Angela Leeming, R. J. Zhang, Dayoung Jung, Hannah Shi, Hadley Grundman, Darian Doakes, Kathleen Cui, Changying Jiang, Makhdum Ahmed, Krystle Nomie, Bingliang Fang, Michael Wang, Yixin Yao, Liang Zhang

**Affiliations:** 1 Department of Lymphoma and Myeloma, The University of Texas MD Anderson Cancer Center, Houston, TX USA; 2 Department of Thoracic Surgery, The University of Texas MD Anderson Cancer Center, Houston, TX USA; 3 Department of Biostatistics, The University of Texas MD Anderson Cancer Center, Houston, TX USA; 4 Department of Stem Cell Transplantation and Cellular Therapy, The University of Texas MD Anderson Cancer Center, Houston, TX USA

**Keywords:** B-cell lymphoma, Drug development, Cancer therapeutic resistance

Dear Editor,

B-cell lymphomas such as relapsed or refractory mantle cell lymphoma (MCL) and diffuse large B-cell lymphoma (DLBCL) are aggressive non-Hodgkin lymphomas (NHL). Although the BTK inhibitor ibrutinib has offered markedly improved clinical outcomes after disease relapse from multiple prior therapies^1,2^, ibrutinib resistance often develops in MCL, even following initial positive responses, and DLBCL is frequently resistant to ibrutinib^2,3^. We and others have found that specific tumor suppressor gene defects are correlated with relapsed/refractory characteristics and cause poor clinical outcomes^4,5^. However, these specific tumor suppressors, including *TP53* and *CDKN2A*, are not druggable, and indirectly targeting these tumor suppressor-mediated pathways has not resulted in high clinical response rates. Auranofin, a gold-containing compound that is FDA-approved for treatment of rheumatoid arthritis, is being repurposed as a potential anti-tumor drug against different refractory malignancies^6^. Currently, auranofin is being assessed in clinical trials for chronic lymphocytic leukemia, breast cancer, and lung cancer (NCT01747798, NCT01419691, and NCT01737502, respectively). Auranofin has a proven safety profile, making it an attractive compound for clinical trials^7^, as an estimated 70–90% of agents fail clinical trials, with safety concerns being a major cause of drug development discontinuation^8^. However, repurposing FDA-approved drugs with known safety in humans for new indications may circumvent these issues and offer a promising new area of investigation. Here, we demonstrate that auranofin targets thioredoxin reductase-1 (Txnrd1) to effectively induce DNA damage, reactive oxygen species (ROS) production, cell growth inhibition, and apoptosis in aggressive B-cell lymphomas, especially in *TP53*-mutated or *PTEN*-deleted lymphomas. First, auranofin has consistently shown to induce lethality in a panel of DLBCL and MCL cell lines. We treated 8 DLBCL cell lines—including three GCB-type cell lines (OCI-Ly8, OCI-Ly7, and Su-DHL-10) and five ABC-type cell lines (OCL-Ly3, OCI-Ly10, U2932, TMD8, and HBL-1)—and six MCL cell lines (Z-138, JVM-2, Mino, Maver-1, Jeko-1, and Jeko-R) with auranofin in concentrations ranging from 0 to 5 μM for 72 h and tested cell viability using a luminescent assay. We found that auranofin was cytotoxic to both DLBCL (Fig. 1a) and MCL (Fig. 1b) in a dose-dependent manner, with an IC_50_ range of 0.058–1.389 μM. *TP53*-mutated lymphoma cells, Mino, Maver-1, OCI-Ly7, OCI-Ly8, Su-DHL-10, U2932, JeKo-1, and JeKo-R, as well as *PTEN*-lost Z-138 were much more sensitive to auranofin. However, the *TP53*-intact lymphoma cell lines OCI-Ly3 and JVM-2 had much higher IC_50_ values than the other cell lines (Fig. 1c). The JeKo-R cell line consists of ibrutinib-resistant MCL cells established by long-term exposure to an escalating dose of ibrutinib during JeKo-1 cell culture, and it represents acquired resistance to ibrutinib. Z-138 has intrinsic resistance to ibrutinib due to its constitutive NIK signaling activation^9^. Our data demonstrate that the IC_50_ of auranofin in Z-138 is only 0.058 μM, which is 10-fold lower than the IC_50_ of the *PTEN*- or *TP53*-intact lymphoma cell lines (Fig. 1c).

**Fig. 1:**
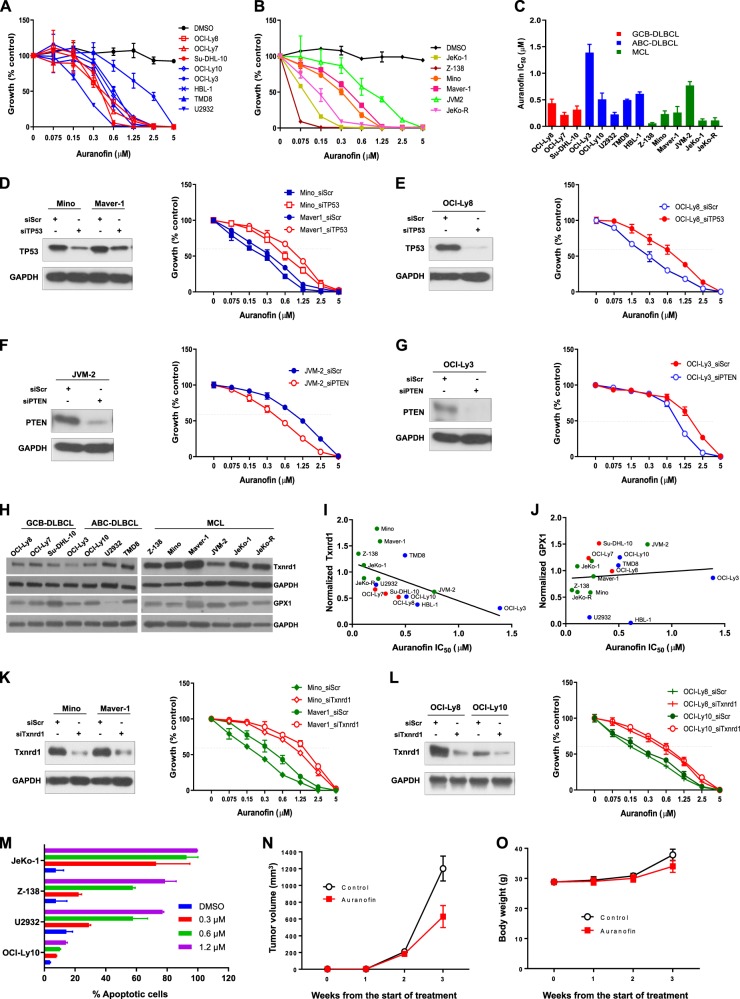
Auranofin-induced cytotoxicity to lymphoma cells is related to Txnrd1 but not GPX1. **a** Dose-response curve for DLBCL treated with auranofin for 72 h. Red = GCB type; Blue = ABC type. **b** Dose-response curve for various MCL cell lines treated with auranofin for 72 h. **c** Averaged auranofin IC_50_ calculated from repeated non-linear regression of dose-response curves. **d** Mino, Maver-1, and **e** OCI-Ly8 cell lines were immunoblotted by anti-TP53 antibody after 48-h knockdown of TP53 by siRNA (siTP53) and scrambled siRNA (siScr), and auranofin-treated dose-response growth curves were shown at the presentence of siTP53 or siScr for 72 h. **f** JVM-2 and **g** OCI-Ly3 cell lines were immunoblotted by anti-PTEN antibody after 48-h knockdown of PTEN by siRNA (siPTEN) and siScr, and auranofin-treated dose-response growth curves were shown at the presentence of siPTEN or siScr for 72 h. **h** Immunoblot of Txnrd1 and GPX1 in MCL and DLBCL. **i** Txnrd1 protein level, and **j** GPX1 protein level to the relation with IC_50_ of auranofin in DLBCL and MCL cell lines. A Pearson’s correlation test was performed with *p* < 0.05 being treated as significant. **k** Mino, Maver-1, and **l**. OCI-Ly8 and OCI-Ly10 cell lines were immunoblotted by anti-Txnrd1 antibody after 48-h knockdown of Txnrd1 by siRNA (siTxnrd1) and siScr, and auranofin-treated dose-response growth curves were shown at the presentence of siTP53 or siScr for 72 h. **m** Auranofin-induced apoptosis at 24 h as measured by Annexin-V binding assay. **n** The in vivo effects of auranofin in *TP53*-mutated DLBCL PDX model. Mice were administered vehicle control or auranofin 50 mg/kg, oral gavage, daily for 21 consecutive days after 3 days of tumor engraftment. Tumor burden was calculated by measuring tumor volume (*n* = 5; auranofin vs. vehicle, *p* = 0.000213). **o** Body weight was calculated during drug treatment (auranofin vs. vehicle, *p* = 0.01556).

To address the correlation of auranofin and *TP53* mutation and *PTEN* loss, in *TP53*-mutated MCL cell lines Mino, Maver-1 (Fig. 1d), and DLBCL cell line OCI-Ly8 (Fig. 1e), *TP53* knockdown made cells more tolerant to auranofin treatment (each pair cell lines siTP53 vs siScr, *p* < 0.0001). However, *TP53* knockdown in two *TP53* wild-type cell lines JVM-2 and OCI-Ly3 did not affect the changes of growth inhibition after auranofin treatment (data not shown), indicating that only mutated-TP53 sensitized cells to auranofin. Next, we specifically knocked down *PTEN* in JVM-2 (Fig. 1f) and OCI-Ly3 (Fig. 1g), cells became more sensitive to auranofin treatment (each pair cell lines siPTEN vs siScr, *p* < 0.0001). The result is consistent with the data in *PTEN*-lost Z-138 cells (Fig. 1c) indicating PTEN loss sensitized cells to auranofin treatment.

A recent publication shows that the main function of auranofin is to inhibit Txnrd1 in the cytoplasm and nucleus to induce ROS production, tumor cell growth inhibition, and apoptosis^10^. Txnrd1 could therefore be the therapeutic target of auranofin for lymphoma. In addition, *TP53* and *PTEN* regulate glutathione perosidase-1 (GPX1), which results in ROS accumulation and cell damage^11^. Therefore, we investigated the expression of both Txnrd1 and GPX1 in lymphoma cells, especially *TP53*-mutated or *PTEN*-deleted lymphoma cells, and explored their correlation with auranofin treatment. We found that all 14 tested aggressive lymphoma cell lines expressed Txnrd1 and GPX1, but the *TP53*-mutated cell line U2932 had very low GPX1 protein levels (Fig. 1h). We used a Pearson’s correlation to evaluate the correlation between Txnrd1 or GPX1 protein levels with the auranofin IC_50_. We found a significant inverse correlation between Txnrd1 and the auranofin IC_50_ (Fig. 1i, *p* = 0.036). However, GPX1 protein levels did not correlate with auranofin IC_50_ (Fig. 1j, *p* = 0.7168). The results indicate that auranofin-induced cytotoxicity to lymphoma cells is related to Txnrd1 protein levels but not to GPX1. To further address the correlation of auranofin to Txnrd1, Txnrd1 knockdown in Mino, Maver-1 (Fig. 1k), and OCI-Ly8 and OCI-Ly10 (Fig. 1l) caused cells more tolerant to auranofin treatment (each pair cell lines siTxnrd1 vs siScr, *p* < 0.0001).

Next, to investigate how auranofin induces apoptosis, we treated the *TP53* or *PTEN* wild-type/mutated/deleted cell lines with 0–1.2 μM auranofin for 24 h, and a dose-dependent apoptosis was observed in all of these cell lines. We observed that the *TP53*-mutated cell lines U2932 and JeKo-1 and the *PTEN*-lost cell line Z-138, were much more sensitive to auranofin treatment than the *TP53* and *PTEN* wild-type cell line OCI-Ly10 (Fig. 1m). To validate the therapeutic effect of auranofin in vivo, we established a DLBCL patient-derived xenograft (PDX) model using freshly isolated tumor cells from a *TP53*-mutated DLBCL patient sample. We found that auranofin significantly inhibited tumor growth during 21 consecutive days of 50 mg/kg auranofin treatment by oral gavage (Fig. 1n, *p* = 0.000213). All mice were in good body condition during treatment, and there was no body weight difference between the vehicle and auranofin treatment cohorts (Fig. 1o, *p* = 0.1556).

To elucidate the mechanism of action of auranofin in lymphoma cells, we first showed that auranofin completely inactivated Txnrd1 activity in vitro (Fig. 2a). Two-hour pretreatment with 5 mM antioxidant N-acetylcysteine (NAC), which is a ROS inhibitor, significantly reduced the production of ROS as shown by a significant increase of H2DCFDA fluorescence (Fig. 2b, c, all *p* < 0.0001). To probe the mechanism of auranofin-induced cell death, cells were then treated with auranofin and co-incubated with either 5 mM NAC or 20 μM Z-VAD-FMK, a pan-caspase inhibitor. Incubation with either NAC or Z-VAD-FMK abrogated auranofin-induced apoptosis (Fig. 2d, *p* < 0.05). The results demonstrate that auranofin induced ROS- and caspase-dependent apoptosis. Furthermore, treatment with 1.2 μM or 2.5 μM auranofin reduced the mitochondrial membrane potential at 12 h (Fig. 2e, *p* < 0.05). Since long-term exposure to high levels of ROS causes DNA damage, we then investigated the effects of auranofin on DNA damage response pathways. We found that auranofin dose-dependently activated CHK2 and γH2A.X, as well as an observed concomitant decrease of MDM2 in Z-138, Mino, OCI-Ly8, and OCI-Ly10, suggesting TP53 release and activation in these *TP53*-intact lymphoma cell lines (Fig. 2f). Next, immunofluorescent staining with anti-γH2A.X confirmed that auranofin-treated Z-138 cells had significantly more phosphorylated H2A.X than control cells (Fig. 2g, *p* = 0.0118) and that 5 mM NAC generally inhibited H2A.X phosphorylation (Fig. 2h, *p* = 0.0211). These results demonstrate that these *TP53*-intact lymphoma Z-138 cells are DNA damaged during ROS-dependent apoptosis induced by auranofin treatment, regardless of *PTEN* loss. Interestingly, *TP53*-mutated JeKo-1 cells had no DNA damage response (Fig. 2f), indicating that auranofin-induced cytotoxicity in *TP53*-mutated JeKo-1 cells is not dependent on the DNA damage pathway activation.

**Fig. 2:**
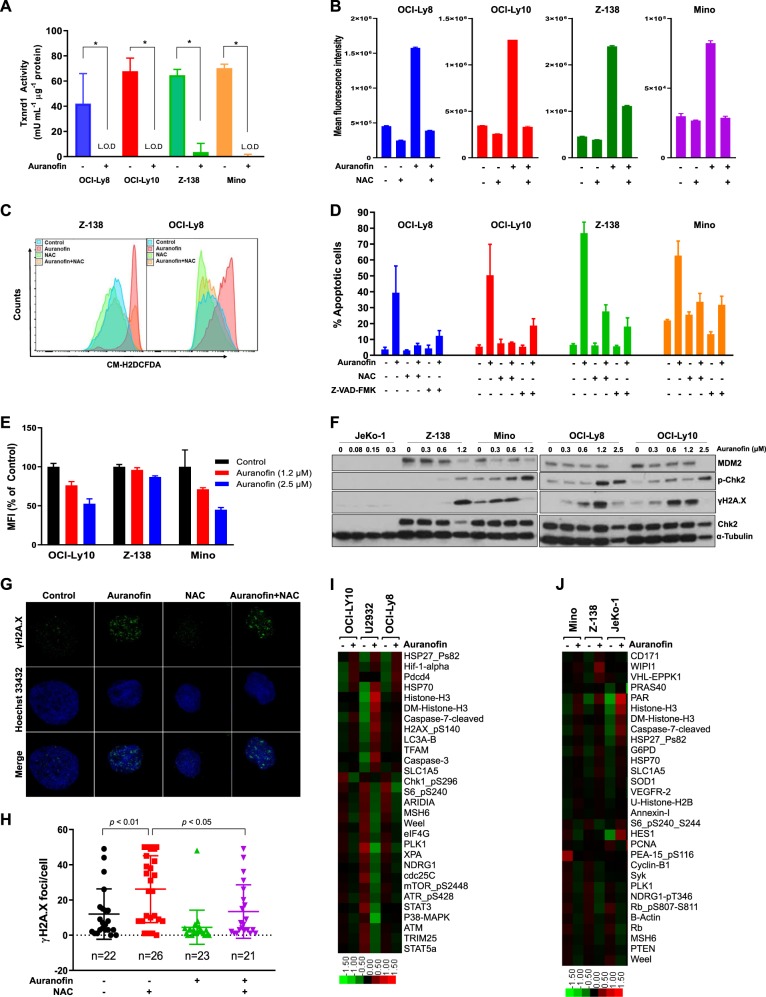
Auranofin inhibits Txnrd1, induces ROS accumulation, DNA damage, cell growth inhibition, and ROS- and caspase-dependent apoptosis in B-cell lymphomas. **a** Lysates from cells treated with 1.2 μM auranofin for 24 h were evaluated for Txnrd1 activity (auranofin vs. vehicle control, two-sample *t*-test, all *p* < 0.05). **b** Pool data and **c** Representative data showed that auranofin significantly increased H2DCFDA fluorescence (two-sample *t*-test, all *p* < 0.0001). Cells were treated with 5 μM auranofin with/without 5 mM NAC for 2 h, and fluorescence was detected by flow cytometry. **d** Cell lines OCI-Ly8, OCI-Ly10, Z-138, and Mino were treated with 1.2 μM auranofin in the presence of either 5 mM NAC or 20 μM Z-VAD-FMK for 24 h. Apoptosis was measured by Annexin-V binding assay. **e** Cells were treated with the indicated doses of auranofin for 12 h and subjected to mitochondrial membrane potential assay (linear regression, all *p* < 0.05). **f** Immunoblot of JeKo-1, Z-138, Mino, OCI-Ly8, and OCI-Ly10 after 24-h auranofin treatment showing DNA damage response. **g** Representative super resolution maximum intensity projections of whole cell micrographs of Z-138 cells treated with 1.2 μM auranofin with/without 5 mM NAC for 24 h. Immunofluorescence staining for γH2A.X and Hoechst is shown. **h** γH2A.X foci were quantitated using the FindFoci ImageJ plugin. At least randomly 20 randomized cells of each treatment condition were evaluated (auranofin vs. vehicle control, *p* < 0.01). NAC prevented auranofin-induced DNA damage (auranofin vs. auranofin plus NAC, *p* < 0.05). **i** DLBCL cell lines OCI-Ly10, U2932, and OCI-Ly8, and **j** MCL cell lines Mino, Z-138, and JeKo-1 were treated with 1.2 μM auranofin for 24 h and then subjected to RPPA analysis. The top 30 most differentially expressed proteins are displayed.

To further investigate other pathways that auranofin targets in both *TP53* wild-type and mutated lymphoma cells, we performed RPPA analysis on OCI-Ly8, OCI-Ly10, Mino, Z-138, U2932, and JeKo-1 cell lines. The top 30 most differentially expressed proteins were analyzed in two independent RPPA data analyses of DLBCL (Fig. 2i) and MCL (Fig. 2j). We found that *TP53*-mutated DLBCL and MCL cells have conspicuous changes of protein expression, which were totally different from *TP53* wild-type cells. Especially, in *TP53*-mutated DLBCL cell line U2932, auranofin increased the expression levels of HSP70, histone H3, caspase-3, 7, p-H2A.X, LC3A, and SLC1A5, and decreased the expression of p-S6, ARID1A, MSH6, Wee1, eIF4G, PLK1, XPA, p-NDRG1, Cdc25C, mTOR, ATR, STAT3, ATM, and TRIM25 (Fig. 2i). In *TP53*-mutated MCL cell line JeKo-1, auranofin increased the expression levels of PAR, histone H3, caspase-7, HSP27, and SLC1A5, and decreased the expression of HES1, p-Rb, and Weel (Fig. 2j). The common pattern is that auranofin increased the H3 and SLC1A5 levels and decreased Wee1 expression in both *TP53*-mutated DLBCL cell line U2932 and MCL cell line JeKo-1. SLC1A5 is a glutamine transporter^12^, and Wee1 regulates DNA damage checkpoints^13^. Auranofin may strongly induce metabolic stress, as evidenced by reducing the mitochondrial membrane potential and then increasing the expression of SLC1A5 as compensation for more nutrient supplements from glutaminolysis. In addition, auranofin increases the pro-autophagic protein LC3A and decreases the proteins for signaling activation of mTOR, STAT3, and the cell cycle in *TP53*-mutated DLBCL cell line U2932, indicating that auranofin may have more mechanisms for treating *TP53*-mutated DLBCL.

In summary, our study demonstrates that auranofin exerts its anti-lymphoma cytotoxic effects through ROS-based therapeutics by targeting Txnrd1. Auranofin induces DNA damage, cell growth inhibition, and ROS- and caspase-dependent apoptosis in aggressive B-cell lymphomas, and it especially shows more significant therapeutic effects on *TP53*-mutated or *PTEN*-deleted lymphomas. Our brief study points out that auranofin may be repurposed as an effective clinical option for *TP53*-mutated or *PTEN*-deleted refractory B-cell lymphoma.

## Supplementary information

Supplemental Materials and Methods

## References

[1] Wang ML (2013). Targeting BTK with ibrutinib in relapsed or refractory mantle-cell lymphoma. N. Engl. J. Med..

[2] Wilson WH (2015). Targeting B cell receptor signaling with ibrutinib in diffuse large B cell lymphoma. Nat. Med..

[3] Cheah CY (2015). Patients with mantle cell lymphoma failing ibrutinib are unlikely to respond to salvage chemotherapy and have poor outcomes. Ann. Oncol..

[4] Karube K (2018). Integrating genomic alterations in diffuse large B-cell lymphoma identifies new relevant pathways and potential therapeutic targets. Leukemia.

[5] Eskelund CW (2017). TP53 mutations identify younger mantle cell lymphoma patients who do not benefit from intensive chemoimmunotherapy. Blood.

[6] Fiskus W (2014). Auranofin induces lethal oxidative and endoplasmic reticulum stress and exerts potent preclinical activity against chronic lymphocytic leukemia. Cancer Res..

[7] Heuer MA, Pietrusko RG, Morris RW, Scheffler BJ (1985). An analysis of worldwide safety experience with auranofin. J. Rheumatol..

[8] Woodcock J, Woosley R (2008). The FDA critical path initiative and its influence on new drug development. Ann. Rev. Med..

[9] Rahal R (2014). Pharmacological and genomic profiling identifies NF-kappaB-targeted treatment strategies for mantle cell lymphoma. Nat. Med..

[10] Lee D (2018). Induction of oxidative stress via inhibition of thioredoxin reductase 1 is an effective therapeutic approach for hepatocellular carcinoma. Hepatology.

[11] Wu Y (2014). PTEN phosphorylation and nuclear export mediate free fatty acid-induced oxidative stress. Antioxid Redox Signal.

[12] van Geldermalsen M (2016). ASCT2/SLC1A5 controls glutamine uptake and tumour growth in triple-negative basal-like breast cancer. Oncogene.

[13] Geenen JJJ, Schellens JHM (2017). Molecular pathways: targeting the protein kinase Wee1 in cancer. Clin. Cancer Res..

