# Supplementary material for: Repurposing auranofin to treat TP53-mutated or PTEN-deleted refractory B-cell lymphoma
Source: Blood Cancer J. 2019 Nov 28;9(12):95. doi: 10.1038/s41408-019-0259-8 (PMC6882812; doi:10.1038/s41408-019-0259-8)
Supplement: Supplementary file 1 — Supplemental Materials and Methods [file 41408_2019_259_MOESM1_ESM.docx]

**Materials and Methods**

*Cell lines and cell culture*

MCL cell lines Z138, Mino, JVM-2, Maver-1, Jeko-1, and Jeko-R were purchased from the American Type Culture Collection (Manassas, VA, USA). Cells were cultured in RPMI-1640 media supplemented with 10% fetal bovine serum and 1% penicillin-streptomycin. DLBCL cell lines OCI-Ly3, OCI-Ly10, OCI-Ly7, OCI-ly8, Su-DHL10, TMD8, HBL-1, and U2932 were kindly gifted by Dr. Richard Davis in the Department of Lymphoma and Myeloma at MD Anderson Cancer Center (Houston, TX, USA). OCI cell lines were cultured in IMDM supplemented with human plasma, and others were cultured in RPMI-1640 supplemented with 15% fetal bovine serum. All cell lines were fingerprinted and mycoplasma tested by the MD Anderson core facility.

*Reagents and antibodies*

Auranofin powder was dissolved in DMSO as a 1000 × stock (Sigma-Aldrich, St. Louis, MO, USA). N-acetyl cysteine (NAC) was dissolved in water and pH adjusted to pH 7.0 with NaOH. Z-VAD-FMK caspase inhibitor was prepared as a 20 mM stock in DMSO (R&D Systems, Minneapolis, MO, USA). MDM2, p-CHK2, CHK2, γH2A.X, PTEN, and GAPDH antibodies were purchased from Cell Signaling Technology (Danvers, MA, USA). Txnrd1, TP53 and α-Tubulin antibodies were purchased from Santa Cruz Biotechnology (Dallas, TX, USA), and GPX1 antibody was purchased from Abcam (San Francisco, CA, USA).

*Cell viability assay*

Cells were seeded in triplicate into 96-well plates (5000 - 7500 cells/well) and treated with varying concentrations of auranofin with DMSO as a negative control for 72 hours. In the last 30 minutes, 50 µL of CellTiter 96 Aqueous One Solution Reagent (Promega, Madison, WI, USA) were added to the culture wells and incubated at 37 ^o^C in 5% CO_2_. Light absorbance of formazan was measured at 495 nm on a universal microplate reader equipped with KC4 software (BioTek Instruments, Winooski, VT, USA).

*Annexin V binding assay*

Cells were plated in 6-well plates at a density of 0.2 ×10^5^/mL and treated with 0, 0.15, 0.3, 0.6, 1.25, or 2.5 μM auranofin with/without the exposure of 5mM NAC or 20 μM Z-VAD-FMK for 24 hours. Cells were harvested and washed with Phosphate Buffered Solution (PBS) then stained with propidium iodide and annexin-V-FITC (BD Biosciences, San Jose, CA, USA). Flow cytometric data were collected on a Novocyte flow cytometer (Acea Biosciences, Inc., San Diego, CA, USA) and analyzed using FlowJo software.

*siRNA mediated knockdown of TP53, PTEN and TXNRD1*

MCL and DLBCL cell lines were grown in respective growth media overnight to reach log phase of growth, before they were seeded at 4x10^6^ cells/well in 6-well tissue culture plates. The cells were transfected with control and target specific siRNA to achieve a working concentration of 50nM using Liofectamine2000 (Invitrogen). For proliferation assay, siRNA transfected cells were seeded in triplicate at 5000-7500/well into 96-well plates containing Auronafin at indicated concentrations. Cell viability assay was performed as described above.

Silencer pre-designed target specific and negative control siRNA were purchased from Life Technology: siRNA_PTEN_ID114050 (Sense 5’-CGAACUGGUGUAAUGAUAUtt-3’; anti-sense 5’-AUAUCAUUACACCAGUUCGtc-3’); siRNA_TP53_ID605 (Sense 5’-GUAAUCUACUGGGACGGAAtt -3’; anti-sense 5’- UUCCGUCCCAGUAGAUUACca-3’); siRNA_ Txnrd1 _IDs755 (Sense 5’-GGUUUACCAUAGUUACUUUtt -3’; anti-sense 5’-AAAGUAACUAUGGUAAACCtc -3’). To monitor target gene expression, treated cells were harvested 48 hours after transfection, total proteins extracted and subjected to Western blotting analysis.

*Western blot*

One million cells from each sample were washed with cold PBS and then lysed using RIPA buffer (Thermo Fisher Scientific, Waltham, MA, USA). Cell lysates were kept on ice for 30 minutes and centrifuged at 14,000 × g for 20 minutes at 4 °C. Supernatants were collected, and the protein content of each fraction was determined using the Bio-Rad Bradford assay (Hercules, CA, USA). After electrophoresis, the proteins were transferred onto a nitrocellulose membrane (Bio-Rad), which was blocked for 2 hours in 5% nonfat dry milk in TBS containing 0.05% Tween-20 ^1^. Proteins were then incubated with the specific antibodies. The membranes were visualized using the electrochemiluminescent detection reagent (Pierce Biotechnology, Rockford, IL, USA).

*ROS and mitochondrial membrane potential FACS assay*

About 2 × 10^6^ cells were harvested and incubated in PBS containing 7.5 μM 6-chloromethyl-2',7'-dichlorodihydrofluorescein diacetate, acetyl ester (CM-H2DCFDA) (Invitrogen, Carlsbad, CA, SUA) for 30 minutes under normal cell culture conditions. The dye-loaded cells were centrifuged, and the supernatant was discarded. Cells were then plated in 6-well plates at a density of 0.5 × 10^6^ cells/mL and pre-treated with 5 mM NAC for two hours. After pre-treatment, auranofin was added at the final concentration of 5 μM. ROS production was labeled with FITC fluorescence after four hours of auranofin treatment and detected by flow cytometry. To evaluate mitochondrial membrane potential, cells were treated in a 6-well plate at a density of 0.5 × 10^6^ cells/mL with either 1.2 μM or 2.5 μM auranofin for 12 hours. Then, mitochondrial membrane potential was evaluated using flow cytometry and a tetramethylrhodamine, ethyl ester (TMRE) dye-based assay following the provided protocol (Abcam).

*Reverse phase protein array*

Five × 10^6^ cells were treated with 1.2 μM of auranofin or vehicle control for 24 hours. The cells were then washed and pelleted before submission to the MD Anderson Cancer Center RPPA Core Facility [NCI **# CA16672**]. Raw data were then processed using Cluster 3.0, and heat maps were generated using Java Treeview.

*Immunofluorescence microscopy*

A total of 1 × 10^6^ cells were treated with 1.2 μM auranofin or vehicle control for 24 hours in the presence or absence of 5 mM NAC. The cells were then attached to a poly-l-lysine coated slide before fixation in 4% paraformaldehyde in Dulbecco's phosphate-buffered saline (DPBS) for 10 minutes. The cells were then permeabilized in DPBS with 0.1% Triton X-100 for 10 minutes before being blocked for one hour in 5% bovine serum albumin in DPBS with 0.3% Tween-20. The cells were incubated with γH2A.X antibody overnight, then washed and incubated with Alexa-488 conjugated antibody and 0.2 μg/mL of Hoechst 33432 DNA counter stain for one hour. Images were acquired on an OMX Blaze super resolution microscope. Maximum intensity projects were then used to be processed by the FindFoci ImageJ plugin ^2^.

*DLBCL PDX model*

DLBCL PDX model was established as described previously ^3^. The specimen obtained from patient with relapsed DLBCL was informed consent and approved by the Institutional Review Board at The University of Texas MD Anderson Cancer Center. All experimental procedures and protocols were approved by the Institutional Animal Care and Use Committee of The University of Texas MD Anderson Cancer Center. In Brief, 10-week-old male NSG mice (Jackson Laboratory) were housed in the animal research facility. Five × 10^6^ freshly isolated DLBCL cells from a TP53-deleted clinical sample were directly injected into bone chip of NSG-hu mice after the mice were anesthetized with 5% isoflurane vaporizer. Once tumor growth was detected in the first generation, tumor mass was monitored and then passaged. The mice that equally growing tumor were randomly assigned as 5 mice/group (n= 5) for in vivo treatment. Three days after tumor implantation, the mice were administered vehicle control or auranofin 50mg/kg, oral gavage, daily for 21 consecutive days. Tumor burden was evaluated by measuring tumor volume using a formula volume (v) = a × b^2^/2 (a: long diameter; b: short diameter). Survival time was calculated from treatment day to endpoint (one diameter of tumor mass reaches 15 mm or when mouse become moribund).

*Statistical analysis*

All assays were performed in triplicates and expressed as mean values ± SEM or SD. A non-linear fit for dose-response with repeated measurements was used to determine IC50. The strength of IC50 correlation with Txnrd1 and GPX1 was tested using a Pearson’s correlation test. Direct comparisons between treated groups were conducted using Student’s t-test or Wilcoxon rank sum test. Linear regression with proper transformation was applied to examine the dose effects when multiple doses were tested. Overall survival was measured using the Kaplan-Meier method. *P* values of less than 0.05 were declared as statistically significant. All analyses were performed with software *R* v3.4.1 and *GraphPad Prism* v7.03.

**References:**

1 Mai, Y. *et al.* An oxidative stress-based mechanism of doxorubicin cytotoxicity suggests new therapeutic strategies in ABC-DLBCL. *Blood* **128**, 2797-2807, doi:10.1182/blood-2016-03-705814 (2016).

2 Herbert, A. D., Carr, A. M. & Hoffmann, E. FindFoci: A Focus Detection Algorithm with Automated Parameter Training That Closely Matches Human Assignments, Reduces Human Inconsistencies and Increases Speed of Analysis. *PLOS ONE* **9**, e114749, doi:10.1371/journal.pone.0114749 (2014).

3 Zhang, L. *et al.* B-Cell Lymphoma Patient-Derived Xenograft Models Enable Drug Discovery and Are a Platform for Personalized Therapy. *Clin Cancer Res* **23**, 4212-4223, doi:10.1158/1078-0432.CCR-16-2703 (2017).
